# Development of Green and Sustainable Cellulose Acetate/Graphene Oxide Nanocomposite Films as Efficient Adsorbents for Wastewater Treatment

**DOI:** 10.3390/polym12112501

**Published:** 2020-10-27

**Authors:** Ali Aldalbahi, Mehrez El-Naggar, Tawfik Khattab, Meram Abdelrahman, Mostafizur Rahaman, Abdulaziz Alrehaili, Mohamed El-Newehy

**Affiliations:** 1 Department of Chemistry, College of Science, King Saud University, Riyadh 11451, Saudi Arabia; mrahaman1997@gmail.com (M.R.); 436107406@student.ksu.edu.sa (A.A.); melnewehy@ksu.edu.sa (M.E.-N.); 2 Textile Research Division, National Research Centre, Giza 12311, Egypt; mehrez_chem@yahoo.com (M.E.-N.); tkhattab@kent.edu (T.K.); abdelrahman.meram@yahoo.com (M.A.)

**Keywords:** sugarcane bagasse, cellulose acetate, graphene oxide, adsorbent, Ni^2+^, kinetics, nanocomposite, water treatment

## Abstract

Novel ecofriendly adsorbents, cellulose acetate/graphene oxide (CA-GO) nanocomposite, were prepared from sugarcane bagasse agro-waste for removing Ni^2+^ ions from wastewater. Graphene oxide (GO) was prepared by the oxidation of sugarcane bagasse using ferrocene under air atmosphere. Cellulose acetate (CA) was also prepared from sugarcane bagasse by extraction of cellulose through a successive treatments with sulfuric acid (10% v/v), sodium hydroxide (5% w/v), ethylenediaminetetraacetic acid, and hydrogen peroxide, and finally, followed by acetylation. CA-GO was prepared via mixing of GO and CA in the presence of calcium carbonate and different concentrations of GO, including 5, 10, 15, 20, 25, and 30 wt% relative to the weight of CA. The CA-GO nanocomposite showed porous microstructures with high surface area, which enhance their ability towars the adsorption of Ni^2+^ ions from wastewater. The morphological properties of the prepared adsorbents were explored by scanning electron microscope (SEM) and Fourier-transform infrared spectroscopy (FT-IR). The efficiency of the CA-GO towards the adsorption of Ni^2+^ ions from wastewater was explored against as time, temperature, and total content of Ni^2+^ ions. The adsorption measurements of Ni^2+^ ions were investigated within the concentration range of 10–40 mg/L, time range between 15 and 90 min, and temperature range between 25 °C and 55 °C. The results displayed a considerable improvement in the adsorption process of Ni^2+^ ions by CA-GO-2 with a removal efficiency of 96.77%. The isotherms were monitored to best fit the Langmuir model. Finally, the adsorption performance of the prepared CA-GO nanocomposite films demonstrated promising properties as green, sustainable and cheap adsorbents for water pollutants.

## 1. Introduction

Agricultural wastes are significant source for raw materials for the green and sustainable manufacturing of various industrial products and alternative resources of chemicals, polymers, materials, and energy [1,2,3,4,5,6,7,8]. The utilization of agro-wastes, such as rice straw, corn strove and ugarcane bagasse, as a feedstock has not suffered from the wide price fluctuations. The use of green and sustainable composites prepared from agro-wastes has presented numerous advantages such as low energy consumption, renewability, biodegradability, biocompatibility, low density low cost compared to synthetic materials [9,10,11,12,13].

Cellulose acetate (CA) is biopolymer that can be employed in various fields such as gas separation, packaging, and water purification. CA is characterized by simple molecular structure and excellent properties such as biodegradability, simple processing, accessibility from different renewable resources, high transparency, beneficial physical properties, and unique mechanical properties [9,10,11,12,14].

On the other hand, graphene has limited applications in water purification, which can be overcame by generating some substituents onto graphene-based composite leading to increase of its environmental applications. Graphene has been utilized to produce graphene oxide (GO), which can be simply dispersed in various solvents [15,16]. Graphene oxide sheets have been utilized in the preparation of strong paper-like thin films, membranes, and other composite materials [17,18] as well as nonlinear optical materials, optical lenses, and energy conversion devices [19,20,21]. 

It is noteworthy that the use of impure water has been one of the major reasons for 2.2 million diarrhea deaths annually, mostly in children. In addition to nasal ulceration, impure water causes liver failure, dermatitis, and cancer, which can be attributed in many cases to toxic heavy metal pollutants in drinking water [22,23]. Therefore, the elimination of heavy metals such as nickel, mercury, cadmium, lead, zinc and copper, from wastewater has high priority due to their highly poisonous and carcinogenic effects, causing damage in different human body organs. Heavy metals can be easily adsorbed by marine organisms’ food chains, resulting in indirect human health risks. Moreover, the ions of heavy metals can enter the human body through either the respiratory or digestive tracts [24,25]. Nickel (Ni)-based compounds are non-biodegradable and toxic [19,20,21,22] and can be easily adsorbed by the lungs into the bloodstream and accumulate in the lungs with time, resulting in health defects such as lung scar tissue, bronchitis, and pulmonary fibrosis, as well as lung cancer and other respiratory tumors. The World Health Organization identified the highest allowed total content of nickel ions in wastewater from the modern electroplating industries to be lower than 4.1 mg/L, and it must be lower than 0.1 mg/L in drinking water [25,26,27,28]. There are various sources from which nickel pollutants enter water streams; the nickel pollutants are generally from industrial processes such as metal finishing, forging, the battery industry, mining, and electroplating [29,30,31,32]. There are various methods that were utilized for removing Ni^2+^ from aqueous environments, such as ion exchange, filtration, chemical precipitation, membrane separation, reverse osmosis, and adsorption, ion exchange, filtration, membrane, reverse osmosis and chemical precipitation [33,34,35]. Among these techniques, the adsorption is a simple and low-cost technique that can be used for removing metal ions. Different natural adsorbents have been utilized to remove nickel ions from wastewater containing waste such as sugar industry waste, tea waste, fly ash, green algae, spent animal bones, activated carbon, aerobic activated sludge, loofah sponge-immobilized biomass of *Chlorella sorokiniana*, crab shell, and seaweed [36,37,38,39]. The low adsorption capacity of such adsorbents limits their applications, but there is a major demand to explore novel, simple, green, and sustainable adsorbents with high adsorption capacity at low cost. To the best of our knowledge, the utilization of sugarcane-bagasse-based adsorbent materials for the adsorption of metal ions, particularly, Ni^2+^, is still extremely limited in the literature. 

Herein, we describe the preparation of environmentally friendly cellulose acetate/graphene oxide (CA-GO) nanocomposite adsorbents, starting from sugarcane bagasse agro-wastes for environmental applications. CA-GO nanocomposite adsorbents with porous microstructures, large surface area, and high adsorption ability were used for removing Ni^2+^ from wastewater. The effects of adsorption parameters, including temperature, time, and initial concentrations of Ni^2+^ on the adsorbent uptake were investigated. Langmuir and Freundlich systems were utilized to fit the results.

## 2. Experimental Details

### 2.1. Materials and Chemicals

Sugarcane bagasse agro-wastes were provided courtesy of Quena Paper Industry, Cairo, Egypt. The sugarcane bagasse was firstly air-dried and homogenized to prevent differences in chemical compositions of different batches. It was then grinded to a mesh size of 450 *μ*. Ferrocene, calcium carbonate (>99%), and hydrochloric acid (37%) were purchased from Sigma-Aldrich, Munich, Germany. All solvents were of analytical grades (Sigma-Aldrich, Munich, Germany.) and were used as received without further purification. 

### 2.2. Synthetic Approaches

#### 2.2.1. Preparation of Graphene Oxide (GO)

GO was prepared by oxidizing sugarcane bagasse with ferrocien at [40], where sugarcane bagasse (2.5 g) and ferrocene (0.5 g) were mixed at 300 °C for 10 min under air atmosphere to generate graphene oxide as a black solid.

#### 2.2.2. Preparation of Cellulose Acetate (CA)

CA was generally prepared using the literature procedure described by Candido et al. [41]. 

First, in a glass beaker (4 L), pretreatment of sugarcane bagasse (1/10 w/v) with sulfuric acid (10% v/v) was performed by stirring the mixture at 100 °C for 40 min in a thermal bath. The solid portion was isolated from the fluid portion by filtration under vacuum, subjected to washing with distilled water till reaching neutral pH value, and air-dried under ambient conditions. The dry solid product (1/10 w/v) was transferred to a polypropylene beaker (4 L) containing sodium hydroxide (5% w/v). The admixture was subjected to stirring in in a thermal bath at 100 °C for 60 min. The solid product was isolated by filtration under vacuum, washed with water till reaching a neutral pH value, and air-dried under ambient conditions. The generated dry solid material (10% w/v) was then subjected to a chelating process in a polypropylene bag at 70 °C for 30 min using ethylenediaminetetraacetic acid (0.5%). The solid material was filtered off under vacuum and washed with distilled water at 70 °C. After chelating, the chemical bleach process was performed at 70 °C for 45 min in a polypropylene bag containing the produced solid material (5% w/v), H_2_O_2_ (5% v/v), and magnesium sulfate (0.1%). The medium pH was attuned at 12 by sodium hydroxide (0.25 M). The generated solid material was filtered-off, washed with water at 70 °C till reaching a neutral pH value, and finally, air-dried under ambient conditions.

CA was prepared by stirring the extracted cellulose (10 g) with glacial acetic acid (24 mL) at 37.8 °C for 60 min. Then, an extra 40 mL of glacial acetic acid and sulfuric acid (0.08 mL) were added to the admixture, with continued stirring for 45 min. After cooling to 18.3 °C, acetic anhydride (28 mL) and H_2_SO_4_ (0.6 mL) were added; and then the admixture was stirred for 90 min at 35 °C. The final product (CA) can be obtained after slowly addition of glacial acetic acid (20 mL) in distilled water (10.0 mL) over 60 min under stirring. The product was finally washed with distilled water till reaching a neutral pH value.

#### 2.2.3. Determining the Degree of Substitution (DS) 

Cellulose acetate (0.1 g) was added to a mixture of NaOH (0.25 M; 5 mL) and ethyl alcohol (5 mL) and the admixture was left overnight to settle out. HCl (0.25 M; 10 mL) was then added to the admixture and was placed to settle out for 30 min. The admixture was subjected to a titration process using a standard aqueous solution of sodium hydroxide (0.25 M) and phenolphthalein indicator. The percentage of acetyl groups % (AG %) was determined using Equation (1):

AG% = {[(V_bi_ + V_bt_) * *μ*_b_ – (V_a_ * *μ*_a_)] * M * 100}/m_CA_ (1)

where V_bt_ (L) is the volume of NaOH_(aq)_ exhausted in titration, V_bi_ (L) is the volume of the added NaOH_(aq)_, V_a_ (L) is the volume of the added HCl_(aq)_, μ_b_ (M) is the sodium hydroxide concentration, *μ*_a_ (M) is the hydrochloric acid concentration, m_CA_ (g) is the weight of CA and M (43 g mol^−1^) is the molar mass of the acetyl group [12,41]. 

#### 2.2.4. Preparation of Cellulose Acetate/Graphene Oxide (CA-GO) Nanocomposite Adsorbents

Cellulose acetate (7.5 g) was dissolved in acetone (300 mL) with stirring for 60 min. Calcium carbonate (20 g) and glycerol (8.5 g) were then added to the above admixture with stirring. Graphene oxide was then added to the admixture at different quantities, including zero, 5, 10, 15, 20, 25, and 30 wt% relative to the weight of CA (i.e., number of grams of graphene oxide per number of grams CA), which were abbreviated as CA-GO-0, CA-GO-1, CA-GO-2, CA-GO-3, CA-GO-4, CA-GO-5, and CA-GO-6, respectively. Each solution was subjected to ultrasonic homogenization, stirred magnetically for an additional 30 min, drop-casted, onto a film applicator, and finally, air-dried for 24 h to afford a film of a certain thickness (1150 *μ*). Calcium carbonate was removed by dissolution through the immersion of film in a distilled water bath, diluted HCl bath, and finally, air-dried, respectively. The yields of the prepared CA-GO composite adsorbents were calculated according to Equation (2):

Yield % = [(Wt_1_ * Wt_2_)/Wt_3_] * 100 (2)

where Wt_1_ is the starting weight of CA, Wt_2_ is the weight of the generated CA-GO, and Wt_3_ is the molecular weight of CA.

### 2.3. Methods and Apparatus

Scanning electron microscopic (SEM) images of the synthesized cellulose acetate/graphene oxide nanocomposite adsorbents were investigated using Quanta FEG-250, Brno, Czech Republic. Fourier transform infrared (FT-IR) spectra were obtained using the KBr disk method with a Unicam Mattson 5000 spectrometer (Milton Keynes, United Kingdom). The determination of the quantity of Ni^2+^ metal ions quantity was carried out using a Perkin-Elmer 3110 atomic absorption spectrometer (Jersey city, NJ, USA).

### 2.4. Adsorption Evaluation of Ni^2+^

Aqueous solutions o Ni^2+^ (15 mg/L; 20 mL) were prepared, and then the adsorbent (20 mg) was added and stirred at different time periods to investigate the effect of adsorption time. The temperature effect on the adsorbent efficiency was explored by changing the temperature between 25 °C, 35 °C, 45 °C, and 55 °C at a constant time period (30 min). The effect of changing the initial concentration of Ni^2+^ was studied at 10, 20, 30, and 40 mgL^−1^ for 30 min at 25 °C. The concentration of Ni^2+^ was identified by atomic absorption spectrometer. The adsorption efficiency (R%) of Ni^2+^ was identified by employing Equation (3):

Removal efficiency (R%) = (*C_0_* − *C_t_*/*C_0_*) * 100 (3)

where *C_0_* and *C_t_* are the initial and final (after adsorption) total content of Ni^2+^, respectively.

### 2.5. Kinetics Measurements

To attain the controlling rate mechanism of the adsorption processes, including chemical reaction and mass transfer, pseudo-first-order as well as second order models were employed to model the kinetic results of Ni^2+^ adsorption onto the surface of CA-GO. Pseudo first/second order equations were utilized to study the obtained results from the time effect on adsorption. The pseudo first-order modeling depended on the postulation that the physi-sorption (physical adsorption), is the rate determining step as demonstrated by Equation (4):

ln [q_e_ − q_t_] = ln q_e_ − K_1_t (4)

where *q_e_* (mg g^−1^) is the adsorbed quantity of Ni^2+^ at equilibrium, *q_t_* (mg g^−1^) is the adsorbed quantity of Ni^2+^at time *t*, and *K_1_* (min^−1^) is the adsorption rate constant for pseudo first order modeling [42]. 

On the other side, pseudo second order modeling depended on the postulation that the chemi-sorption or known by (chemical adsorption), is the rate determining step as demonstrated by Equation (5):

(*t/q_t_*) = (1/*K_2_q_e2_*) + (*t*/*q_e_*) (5)

where *K_2_* (g/ mg/ min) is the adsorption rate constant for pseudo second order modeling. The values of *qe_2_* and *K_2_* were calculated from plotting *t*/*q_t_* versus *t* using slope and intercept. In the chemical adsorption process, Ni^2+^ was monitored to attach to the graphene oxide surface by chemical bond formation (usually covalent bond formation).

### 2.6. Adsorption Isotherms

The adsorption isotherms afford information on the distribution of the adsorbed molecules among both solid and liquid phases at equilibrium (i.e., affinity of adsorbent, surface characteristics, and adsorption mechanism). The regression coefficient (*R^2^*) was utilized to figure out the best fitting isotherms. The adsorption equilibrium results were found to have the best fit with four types of Freundlich and Langmuir isotherm models. Langmuir is the simplest model depending on the assumption that all adsorption sites are equal and independent. The molecules’ capability to bind is independent of adjacent occupied sites [43]. The Langmuir isotherm can be given by Equation (6):

(C_e_/q_e_) = (1/Kq_m_) + (C_e_/q_m_) (6)

where *q_m_* (mg g^−1^) is the highest adsorption efficiency [43].

On the other hand, the Freundlich isotherm illustrates the un-ideal and reversible adsorption (i.e., an unlimited supply of unreacted graphene oxide sites). It favors better representation of heterogeneous systems. Freundlich isotherm can be estimated by Equation (7):

log *q_e_* = log *K_f_* + (1/n) log *C_e_* (7)

where *K_f_* is the removal efficiency of Ni^2+^. The Langmuir model is restricted to monolayer adsorption systems, while the Freundlich model can be utilized in multilayer systems. 

### 2.7. Thermodynamics Measurements 

The reaction rate can be identified from the determined kinetic data. Nonetheless, the reaction variations that may occur during the adsorption process necessitate the determination of the thermodynamic parameters, including entropy (Δ*S*, kJ/mol) enthalpy, Gibbs free energy (Δ*G*, kJ/mol), and (Δ*H*, kJ/mol) changes during adsorption. Those thermodynamic parameters can be calculated from the van’t Hoff equation (Equation (8)):

ln *K_d_* = (Δ*S*/R) − (Δ*H*/RT) (8)

where R (8.314 J/mol/T) is the gas constant and T (K) is the temperature. The distribution coefficient (*K_d_*) onto the surface of graphene oxide can be determined by Equation (9):

K_d_ = (*C_i_* − *C_e_*/*C_e_*) * (V/m) (9)

Gibbs free energy can be calculated by Equation (10):

Δ*G* = −RT ln *K_d_* (10)

Both ΔH and ΔS can be determined from the van’t Hoff plot of lnK versus 1/T using both slope and intercept [44]. 

## 3. Results and Discussion

### 3.1. Preparation of CA-GO Nanocomposite Adsorbents

Ferrocien-based oxidation of sugarcane bagasse afforded graphene oxide as a black solid powder [40]. Cellulose was extracted from sugarcane bagasse agro-wastes by successive steps, including the treatment of sugarcane bagasse with sulfuric acid, sodium hydroxide, ethylenediaminetetraacetic acid, and finally, hydrogen peroxide to afford pure cellulose [12,41]. CA was prepared by reacting the extracted cellulose with glacial acetic acid and sulfuric acid as a catalyst in a two-stage acetylation reaction. The reaction system was then subjected to hydrolysis to generate cellulose acetate with the desirable DS value (2.45–2.50), which allows a better solubility in different solvents as well as good melting characteristics. Those properties facilitate the use of cellulose acetate in various applications [41,45]. The generated cellulose acetate demonstrated a degree of substitution of 2.45–2.50 as well as a percentage of acetyl substituents of 43.5% to be characterized as triacetate functionalities. In the extraction procedure, the high solubility of lignin was very significant to accomplish a high degree of substitution. Lignin contents compete with cellulose in the acetylation process; thus, the high total content of lignin could reduce the generated yield of the acetylation reaction. The degree of substitution value also proved the significance of the low apparent viscosity, which result in an ease of access to the cellulose chains to increase the effectiveness of the activation step in the acetylation reaction. There was a better accessibility of the ethanolic anhydride to the free hydroxyl substituents on the cellulose polymer chains to result in an improvement in the cellulose activation for the second step of the acetylation reaction. The activation step is critical to the preparation of CA with DS ≥ 2.0. To prepare the cellulose acetate/graphene oxide nanocomposite adsorbent, calcium carbonate, glycerol, and graphene oxide were added to a solution of CA in acetone. Graphene oxide was added at different concentrations, including 0, 5, 10, 15, 20, 25, and 30 wt % relative to the weight of CA. The generated solutions were homogenized, drop-casted onto a film applicator, and finally, air-dried under ambient conditions to give films of certain thickness (1150 *μ*). Excess calcium carbonate was washed out the non-porous composite films by immersion in a water bath, and then immersion in a diluted bath of HCl (_aq_), and air-dried under ambient conditions to afford the corresponding dark brown porous composite films. The yields of the produced porous composite adsorbents are shown in Table 1. The yield of CA-GO was found to directly increase with increasing the total content of graphene oxide to reach its highest value at 62% for CA-GO-5. The yield was then found to decrease at higher concentration of graphene oxide (CA-GO-6), which could be ascribed to the super-saturation with graphene oxide. The yield of the pure cellulose acetate (CA-GO-0; yield of 46%) was higher than the sample containing graphene oxide at the lowest concentration (CA-GO-1; yield of 42%).

### 3.2. Characterization of CA-GO

**Figure 1:**
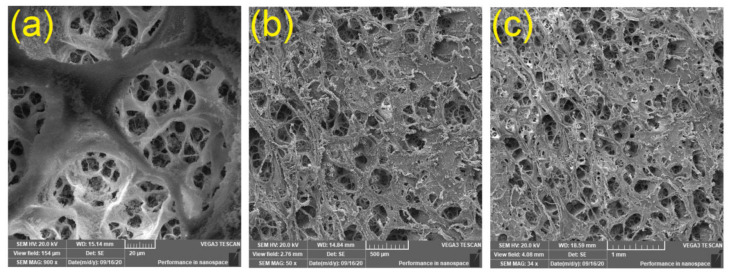
SEM images of CA membranes after washing out CaCO_3_ for CA (**a**) and CA-GO-2 (**b**,**c**).

Adsorption has been commonly used as a simple, practical, highly efficient, and low-cost method for removing Ni^2+^ from wastewater. Therefore, there is a major demand to develop novel, simple, and environmentally benign adsorbents with high adsorption efficiency at low price. Cellulose derivatives have been one of the most significant natural polymers owing to its biocompatibility, renewability, high abundance and biodegradability. Fibrillated cellulose have been used as multifunctional products with microporous structures characterized with high porosity, excellent adsorption, good mechanical properties, high strength-to-weight ratio, high surface area, and low density. CA is a cellulose derivative substituted with multiple acetate groups, which are attached by covalent bonding to the hydroxyl groups of the glucose units on the polymer chains through ester groups. As monitored by SEM images, the average diameters of both pores and fibers increased after washing out CaCO_3_ for the generated film as displayed in Figure 1. 

The produced cellulose acetate/graphene oxide composite films (1150 μm) displayed a sponge-like fibrous morphology. Three-dimensional microporous scaffolds were assembled from CA embedded with graphene oxide. The adsorbents films demonstrated a low density, highly porous structural design, high surface area, and good mechanical characteristics. The surface morphology assessment as well as the distribution of pores was studied by scanning electron microscopic software program, showing an average diameter of 5–15 μm. These characteristics of the fibrillated films resulted in their potential applications as adsorbents in water treatment. The composite films were produced after washing out the water soluble CaCO_3_ away from CA membranes immobilized with graphene oxide to result in porous structures with high porosity and large surface area. In order to wash out CaCO_3_, the composite films were immersed in distilled water. The produced CA-GO films were weighed before and after the removal of CaCO_3_ to indicate the loss of about 93% of CaCO_3_. On the other hand, the water-insoluble graphene oxide will not be washed out of the generated films. The concentration of CaCO_3_ is very high (~265 wt% relative to CA), while, the concentration of graphene oxide is low in the range of 5–30 wt% relative to CA. Additionally, the acetone-soluble graphene oxide was physically immobilized inside within the polymeric strands of cellulose acetate matrix, while the acetone-insoluble CaCO_3_ is physically incorporated inside the cellulose acetate porous system. Therefore, the addition of graphene oxide inside the polymeric strands of the cellulose acetate matrix was found to influence the film microporous system signifying that the graphene oxide was fully immobilized within the composite fibrillated matrix. The number of pores decreased at the cellulose acetate film surface with increasing the concentration of graphene oxide in the polymer matrix. This can be ascribed to the water insoluble graphene oxide which is hard to take away from the cellulose acetate matrix during the washing out process of calcium carbonate by distilled water. Hence, the graphene oxide molecules reduced the number of the formed pores. The graphene oxide molecules located within the polymer strands result in shrinkage of the pore walls, and consequently, result in increasing the diameter of the pore with increasing the graphene oxide concentration. The CA/GO composite films showed high adsorption efficiency to nickel ions, due to its high surface area and high porosity.

**Figure 2:**
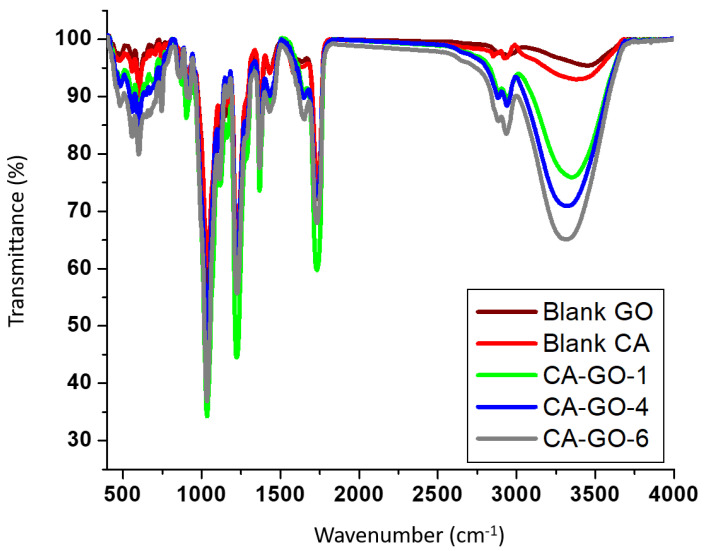
FTIR spectra of graphene oxide powder, blank cellulose acetate film (CA-GO-0), and CA-GO films at lowest total content of GO (CA-GO-1), medium total content of GO (CA-GO-4), and highest total content of GO (CA-GO-6).

FTIR spectra of blank CA and CA/GO composite films as well as graphene oxide powder were presented as shown in Figure 2. The key characteristic absorbance bands of graphene oxide were monitored at 3449, 2940, 1734, 1637, and 1032 cm^−1^, which were attributed to hydroxyl (-OH), aliphatic (CH), carbonyl (C=O), aromatic (C=C), and carboxyl (C-O) substituents, respectively. The key characteristic bands of cellulose acetate were detected at 3388, 2922, 1734, and 1032 cm^−1^, which were attributed to -OH, aliphatic CH, C=O, and carboxylic C-O functional groups, respectively. The prepared CA-GO films showed stretch bands of aliphatic (CH) at 2936 cm^−1^, carbonyl ester of cellulose acetate as well as carboxylic carbonyl of graphene oxide (O-C=O) at 1731 cm^−1^ and hydroxyl (-OH) group at 3313 cm^−1^. The band intensity of the OH groups was found to increase, owing to increasing the concentration of graphene oxide. The aliphatic CH bands of CA-GO films were monitored at 2939 cm^−1^. The -OH stretch bands of CA at 3388 cm^−1^ and GO at 3449 cm^−1^ were shifted to 3345, 3320, and 3313 cm^−1^ for the produced CA-GO-1, CA-GO-4, and CA-GO-6 composite films, respectively. This could be ascribed to the potential hydrogen bond formation between the carboxylic carbonyl group in the graphene oxide molecular system and the -C-OH group in the CA molecular structure. This potential H- bonding brings the CA polymeric chains nearer to each other, and accordingly decreases the thickness of the pore walls, causing an increase in the pore size. Similarly, the carbonyl group was found to shift from 1734 cm^−1^ for both graphene oxide and cellulose acetate to 1731 cm^−1^ for the CA-GO films. The aromatic (C=C) was shifted from 1637 cm^−1^ for graphene oxide to 1648 cm^−1^ for the CA-GO films, which could be ascribed to the reduced stacking between the GO sheets due to better distribution into CA matrix. No other considerable shifts were detected in the positions of the bands with increasing the total content of GO, which denotes that the graphene oxide molecules were completely embedded within the matrix of the cellulose acetate composite film. 

### 3.3. Adsorption Study

#### 3.3.1. Effect of Time 

**Figure 3:**
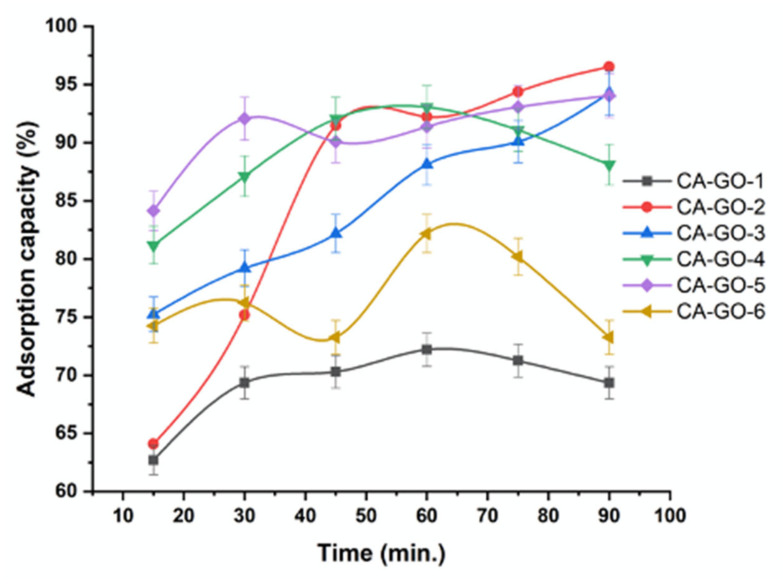
Effect of time on the adsorption of Ni^2+^ onto CA-GO.

The effect of the time on the adsorption efficiency was studied at room temperature (25 °C), an initial concentration of Ni^2+^ of 10 mgL^−1^, and an adsorbent content of 20 mg at different time intervals; 15, 30, 45, 60, 75, and 90 min as shown in Figure 3. The adsorption of Ni^2+^ onto the surface of the adsorbents was rapidly increased in the early stage, due to the high number of active sites; and then the adsorption was increased slowly. No observable adsorptions were monitored after time intervals, including 60, 90, 90, 60, 90, and 60 min, which were mentored as the optimal time intervals for the adsorbents, CA-GO-1, CA-GO-2, CA-GO-3, CA-GO-4, CA-GO-5, and CA-GO-6, respectively. This fact proves that the complex derivatives generated in the initial adsorption stage are unstable, leading to a fast adsorption rate. The next slower adsorption rates could be ascribed to sapping the driving force to result in decreasing the existing adsorption sites as a result of released hydrogen protons to the solution from the oxygen-containing substituents on the adsorbent surface, including hydroxyl and carboxyl groups. The different adsorption efficiencies proved that the absorbents did not exhibit similar morphologies.

#### 3.3.2. Effect of Temperature

**Figure 4:**
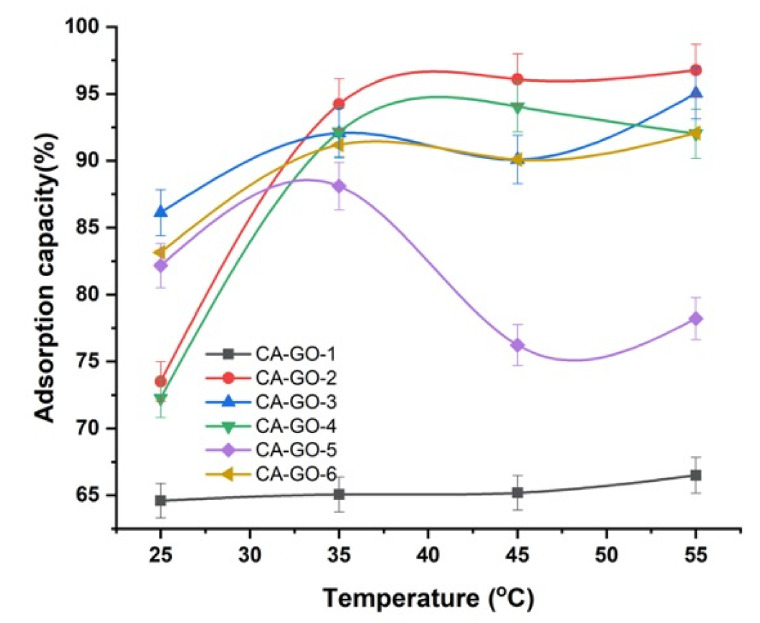
Effect of temperature on the adsorption of Ni^2+^ onto CA-GO.

The adsorption measurements were performed using an adsorbent weight of 20 mg, an initial concentration of 10 mg/L, and time interval at 30 min. With increasing the temperature from 25 °C to 55 °C, the elimination of Ni^2+^, monitored by samples CA-GO-2 and CA-GO-4, was increased, proposing an endothermic adsorption process (Figure 4). This could be attributed to increasing the diffusion rate of Ni^2+^, with increasing the temperature, throughout the porous structure of the CA-GO derivatives. The adsorption process may include both physical and chemical adsorption due to high temperature, which results in increasing the active sites due to bond rupture. Thus, the endothermic adsorption process could be ascribed to increasing the pore diameter and surface activation. However, no significant changes were monitored in the removal of Ni^2+^, with the increase of temperature from 25 °C to 55 °C, using samples CA-GO-1, CA-GO-3, CA-GO-5, and CA-GO-6, suggesting saturation equilibrium between Ni^2+^ and CA-GO-5/6. This could be attributed to a poor adsorption between CA-GO-5/6 and Ni^2+^ involving an exothermic physical adsorption.

#### 3.3.3. Effect of Ni^2+^ Concentration 

**Figure 5:**
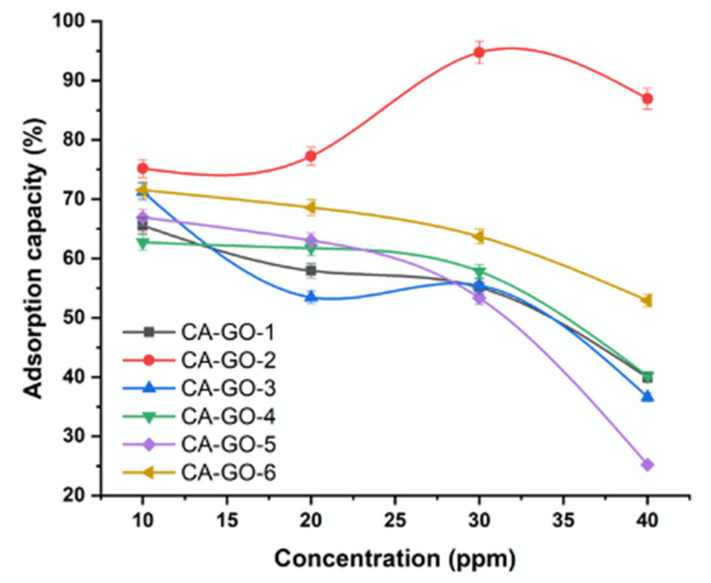
Effect of initial concentration on the adsorption of Ni^2+^ onto CA-GO.

The adsorption measurements were performed at room temperature (25 °C) using different initial concentrations on Ni^2+^ of 10, 20, 30, and 40 mgL^-1^, a time of 30 min, and using 20 mg of CA-GO as demonstrated in Figure 5. The adsorption process increased with increasing the total content of Ni^2+^. It was found that the amount of exchangeable sites in the adsorbents structures and the ratio of Ni^2+^ ions to cellulose acetate are the major reasons for the decreasing of the adsorption with increasing the initial concentrations of Ni^2+^. Upon increasing the ratio of Ni^2+^ ions to GO, the exchangeable sites on GO are saturated, resulting in decreasing the adsorption efficiency. The adsorption capacity of CA-GO-2 was found to increase from 75.18 to 86.94 % with increasing the initial concentrations on Ni^2+^ from 10 to 40 mgL^-1^. This could be ascribed to the considerable driving force transferred by Ni^2+^ concentration to defeat the resistance of mass transfer among solid and liquid phases.

### 3.4. Kinetic Models and Adsorption Isotherms

**Figure 6:**
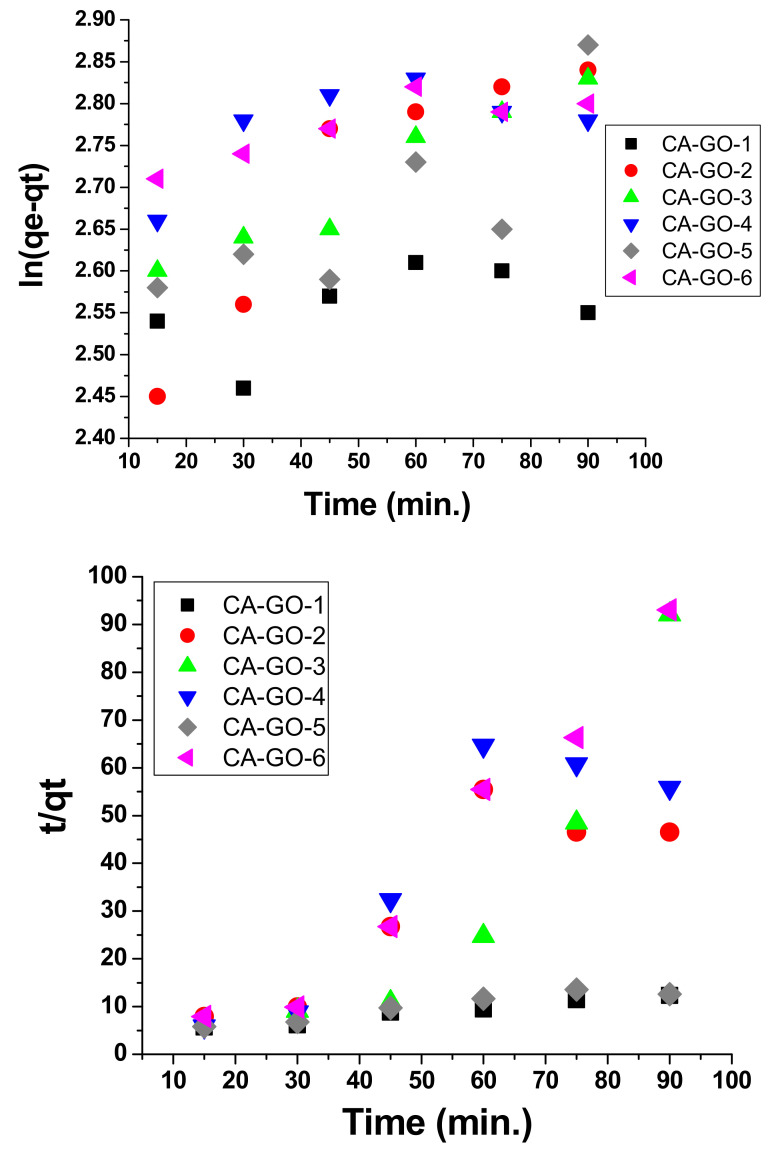
Kinetic models of (top) pseudo first order and (bottom), pseudo second order processes for the adsorption of Ni^2+^ by different CA-GO adsorbents at different time periods.

As shown in Figure 6, pseudo second order modeling demonstrated an improved fit to the adsorption measurements in comparison to pseudo-first-order modeling for all films except CA-GO-3. However, the results accomplished in pseudo-first-order modeling were still appropriate for describing the sorption kinetics of Ni^2+^, indicating that the surface showed both chemi-sorption and physi-sorption adsorption processes by all films except CA-GO-3. The regression coefficient (*R*^2^) of CA-GO-3 demonstrated that pseudo-first-order modeling showed an appropriate fit to the adsorption in comparison to pseudo-second-order modeling. Thus, the surface of CA-GO-3 involved the physi-sorption process.

**Figure 7:**
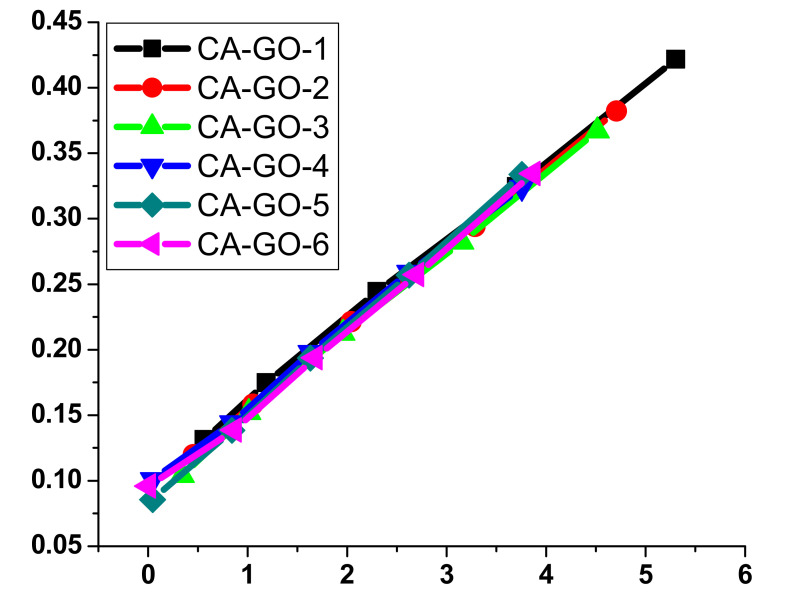

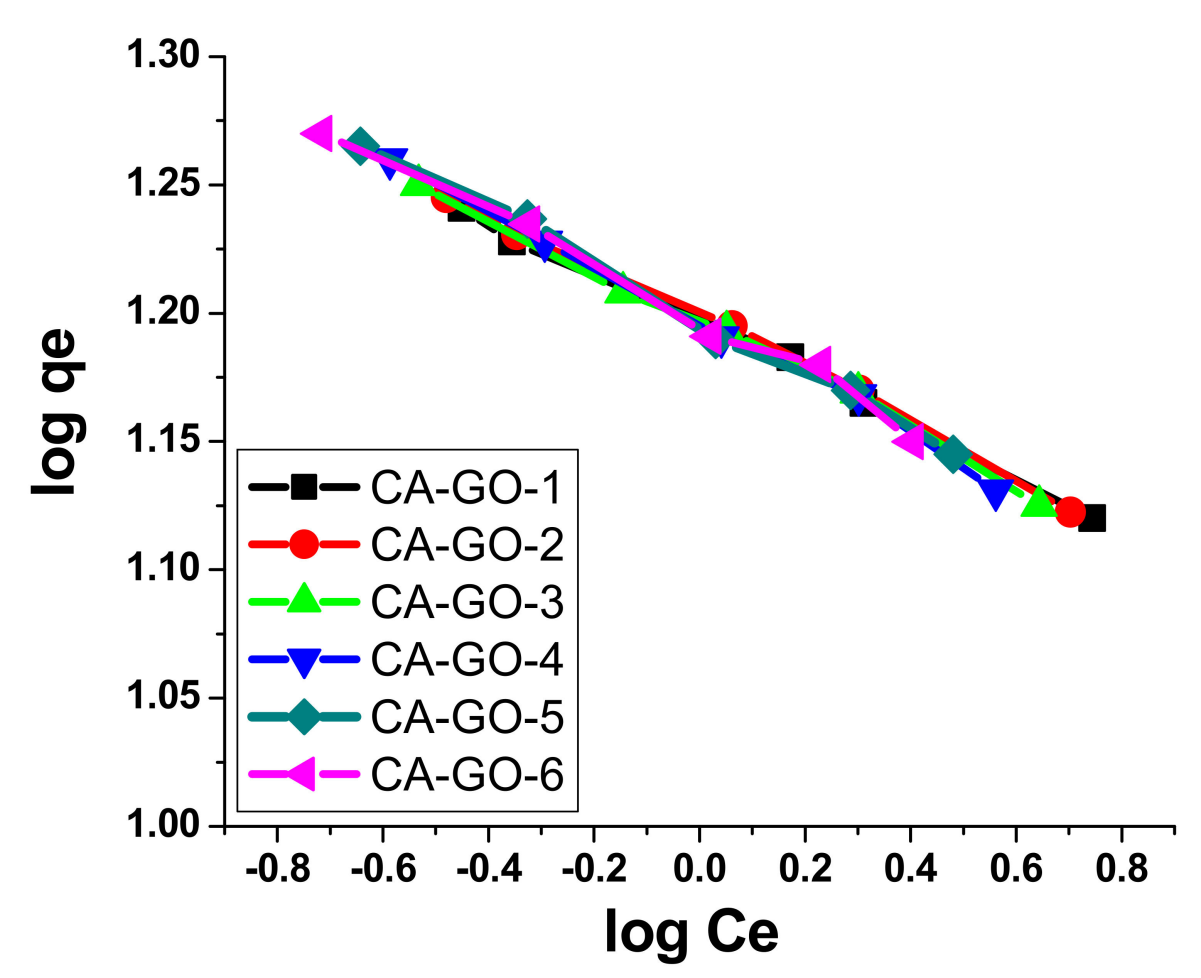
The Langmuir (top) and Freundlich (bottom) adsorption models using different CA-GO adsorbents under ambient conditions (25 °C) for 30 min, CA-GO weight at 20 mg, and initial concentration at 15 mg/L.

Langmuir and Freundlich modeling systems for the adsorption of Ni^2+^ onto CA-GO adsorbents were investigated at room temperature (25 °C) for 30 min using adsorbent weight at 20 mg and initial concentration of 10 mg/L (Figure 7). Isotherms were mainly best fit to Langmuir modeling, which could be attributed to the high regression coefficient (*R*^2^) value (Table 2 and Table 3). Therefore, it can be concluded that the surfaces of all CA-GO adsorbents are homogeneous, and the adsorption occurred mainly in a monolayer system. The thermodynamic measurements for the adsorption process of Ni^2+^ onto the different CA-GO derivatives are displayed in Table 4. Negative Δ*H* values were monitored for CA-GO-2 and CA-GO-3 due to the exothermic reaction, while positive Δ*H* values were monitored for samples from CA-GO-1, CA-GO-4, CA-GO-5, and CA-GO-6 due to the endothermic reaction. The negative Δ*G* indicated a spontaneous sorption. The variations in Δ*S* demonstrated positive values. The increased randomness was monitored during the process of exchanging Ni^2+^. Thus, the adsorption was highly influenced by changing the temperature. 

## 4. Conclusions

The adsorption effeciency of nickel (II) ions from wastewater employing novel eco-friendly derivatives of sugarcane bagasse agro-waste was described. Graphene oxide was prepared by oxidizing of sugarcane bagasse using ferrocene at 300°C under air atmosphere. Cellulose acetate was prepared by an acid–base extraction of cellulose from sugarcane bagasse followed by acetylation. The generated cellulose acetate and graphene oxide were mixed with calcium carbonate to produce novel cellulose acetate/graphene oxide nanocomposite adsorbents using different weight ratios of cellulose acetate to graphene oxide. The developed composite adsorbents demonstrated microporous structures with high surface area and an enhanced adsorption efficiency of Ni^2+^ ions from aqueous media. An increased binding ability of nickel (II) ions was monitored upon increasing the total content of graphene oxide. On the other side, the porosity of the CA-GO composite films was found to decrease with increasing the total content of graphene oxide. Thus, a considerable enhancement in the adsorption capacity by CA-GO-2 was monitored with an adsorption efficiency of 96.77%. The isothermal models were found to best fit the Langmuir model. The adsorption activity demonstrated promising properties as eco-friendly, green, cheap, sustainable, and efficient adsorbents.

## Figures and Tables

**Table 1:** Yields (%) of the generated CA-GO samples.

Sample	Yield (%)
CA-GO-0	46
CA-GO-1	42
CA-GO-2	48
CA-GO-3	53
CA-GO-4	57
CA-GO-5	62
CA-GO-6	34

**Table 2:** Kinetic models of pseudo first order and second order adsorption processes.

Model	Parameter	CA-GO Adsorbent					
		1	2	3	4	5	6
Pseudo first order	*q_exp._*	13.453	17.155	17.402	17.544	15.008	17.327
	*q_calc._*	12.094	15.034	15.124	16.263	13.454	14.826
	*K*_1_	0.363	47 × 10^−4^	32 × 10^−3^	17 × 10^−3^	57 × 10^−4^	18 × 10^−3^
	*R* ^2^	0.635	0.64	0.996	0.774	0.736	0.816
Pseudo second order	*q_calc._*	4.092	0.63	0.883	0.808	3.495	0.727
	*K*_2_	0.759	44 × 10^−2^	43 × 10^−2^	13 × 10^−2^	3.248	62 × 10^−2^
	*R* ^2^	0.902	0.943	0.889	0.795	0.906	0.982

**Table 3:** Models of Langmuir and Freundlich for the adsorption of Ni^2+^ at an initial concentration of 15 mgL^−1^.

Model	Parameter	CA-GO Adsorbent					
		1	2	3	4	5	6
Langmuir	*q_m_* (mg g^−1^)	3.278	12.725	13.986	15.452	12.718	14.541
	*R* ^2^	0.902	0.998	0.998	0.998	0.999	0.997
Freundlich	*K_f_* (mg^(1-1/n)^ g^-1^ L^1/n^)	6.813	3.33	3.335	3.336	3.667	3.665
	*R* ^2^	0.903	0.994	0.995	0.793	0.997	0.817

**Table 4:** Thermodynamic measurements for the adsorption of Ni^2+^.

Parameter		CA-GO Adsorbent					
		1	2	3	4	5	6
Δ*S* (kJ mol^−1^)		0.05	0.07	0.25	0.22	0.22	0.28
Δ*H* (kJ mol^−1^)		−11 × 10^3^	−13 × 10^3^	−62 × 10^3^	−55 × 10^3^	−55 × 10^3^	−65 × 10^3^
ΔG (kJ mol^−1^)	25 °C	−1.781	−2.42	−2.977	−3.822	−2.763	−4.236
	35 °C	−1.78	−4.45	−6.811	−6.833	−8.254	−7.618
	45 °C	−0.972	−2.611	−8.66	−8.222	−8.581	−8.094
	55 °C	−2.209	−4.66	−9.444	−10.076	−9.209	−12.271
